# The ethnobotany of Christ's Thorn Jujube (*Ziziphus spina-christi*) in Israel

**DOI:** 10.1186/1746-4269-1-8

**Published:** 2005-09-28

**Authors:** Amots Dafni, Shay Levy, Efraim Lev

**Affiliations:** 1 Institute of Evolution, University of Haifa, Haifa, 31905, Israel; 2 Dep. of Erets Israel Studies, University of Haifa, Haifa, 31905, Israel

**Keywords:** Israel, ethnobotany, Christ's Thorn Jujube, *Ziziphus spina-christi*, holy tree, sacred tree

## Abstract

This article surveys the ethnobotany of *Ziziphus spina-christi *(L.) Desf. in the Middle East from various aspects: historical, religious, philological, literary, linguistic, as well as pharmacological, among Muslims, Jews, and Christians. It is suggested that this is the only tree species considered "holy" by Muslims (all the individuals of the species are sanctified by religion) in addition to its status as "sacred tree " (particular trees which are venerated due to historical or magical events related to them, regardless of their botanical identity) in the Middle East. It has also a special status as "blessed tree" among the Druze.

## Introduction

Christ's Thorn Jujube (*Ziziphus spina-christi *(L.) Desf. [Rhamnaceae] is a tropical evergreen tree of Sudanese origin. It grows in Israel in all valleys and lowlands, and usually is confined to low elevations below a.s.l. 500 m [1].

The tree and its parts appear to have been in use in Pharaonic industry (carpentry), diet, and in medicine. The fruits were sometimes made into bread, which may also have been used for dressings when warm. Egyptian peasants made similar bread as late as the beginning of the 20 th century [2].

The Christ's Thorn Jujube has been mentioned in classical sources. The Greek botanist. Theophrastus (4^th^–3rd centuries BC) wrote, "The (Egyptian) 'Christ Thorn is more shrubby than the *lotos *(might be *Ziziphus lotus *(l.) Lam.); it has a leaf like the tree of the same name of our country, but the fruit is different, for it is not flat, but round and red, and in size as large as the fruit of the prickly cedar or a little larger; it has a stone which is not eaten with the fruit, as in the case of the pomegranate, but the fruit is sweet, and, if one pours wine over it, they say that it becomes sweeter and that it makes the wine sweeter" [3]. Pliny (1^st ^century AD) mentions the tree in comparison with related species: "The region of Cryonic ranks the lotus below its own Christ-thorn" [4].

This common species is frequently mentioned in Christian as well as Muslim traditions, and was also recorded by pilgrims who visited the Holy Land during generations. We may therefore say that this species is "well soaked" in the local folklore as well as the ethno medicine of almost all the ethnic groups living in the Land of Israel.

Botanists expert in the Bible are constantly engaged in a great debate about what constitutes the "bramble" or "thorns" (Judges 9; 14–15), "thorns" (Matthew 27:27–29) and the "crown of thorn" (John 19:5). Based on local traditions and old sources, today these citations are commonly deemed to refer to *Z. spina-christi *[5-7].

The Quran mentioned the tree twice (LIII: 13–18; LVI: 28–32); the lote-tree is commonly identified as *Z. spina-christi *[8] and accordingly this species is highly respected by the Muslims through the Middle East. This tree has been widely used as a fruit plant and as a medicinal plant since antiquity and is still in use at present.

The aim of this paper is to review the current ethnobotanical status of *Z. spina-christi *in Israel, based on our field studies, in relation to historical and current literature.

## Materials and methods

The field study (1999–2004) centered on Arabic villages in Galilee. Informants were asked about the ritual importance of the plant, why it is respected, which parts are used, and for what purposes. The survey covered 92 informants, consisting of 38 Druze, 54 Muslims (36 Arabs and 18 Bedouins). The informants were mainly chosen according to their knowledge of common traditions and/or religious status. The average age of the informant was 56 years (+/-14 years). The respondents were 90 males and two females (the women were interviewed in the presence of other family members). The question asked was: "What are the significance, uses, traditions, and stories you know about the Christ's Thorn Jujube?" Complementary questions on other trees were introduced only after the informant had expressed his or her view.

The list of medicinal uses during the medieval period was compiled from a survey of written medieval sources [9,10]; the list of medicinal uses of present-day ethnic groups in Israel is based on an ethnobotanical survey [11], an ethnopharmacological survey [12], and other surveys that have been conducted in the Middle East. Medicinal uses mentioned by Palevitch *et al*. [11], which were recorded only from one or two informants, were validated in this survey.

### The plant names

The plant is named *sheisaf *in Hebrew, and a few Bible commentators have identified the tree with the "*atad*" (Job 40:21–22), identified otherwise with *Lycium sp*. [bramble, thorn bush, boxthorn], "*n'atsuts*", and even the "tse'elym" [5,13].

In rabbinical literature, the plant is called *rimin*" (Mishna, Demai, 1:1; Kilayim, 1:4), and in the Talmud it is called "*kanari*" (Bab. Talmud, Baba Bathra, 48b). It may be that it was so named because it is widespread around Lake Kinneret – the Sea of Galilee (Bab. Talmud, Mgillah, 6a).

Several common Arabic names are still in use today: "*nabq*, *dum*, *sidr*, *tsal*, *sadr *[[14-16] and [17]]." *Sidr*" serves as the common name for lotus jujube *Z. lotus*, which is also named "*rubeida*" after its crouch-shaped treetop. The names are used interchancheably in various geographical areas such as Lower Galilee.

In Christian tradition the tree was identified with the thorn bush with which Jesus was crowned before his crucifixion (Matthew 27:28–29; John 19:5; Mark 15:17). This is also the source for the scientific name (*spina-christi*).

The tree is rare in the vicinity of Jerusalem (A. Shmida, personal communication 10 May 2004). But Henry Baker Tristram wrote that he saw a tree in the Kidron valley, outside the city, albeit in the form of a small bush [18]. Tristram gave both the Arabic and the scientific name; so presumably, he was closely familiar with the species. The debate over the identity of the "crown of thorns" in the New Testament is long-lived, and various plants have been suggested as candidates [6,7,19].

#### Islamic sources

The Qur'an says, "And verily he saw him yet another time. By the lote-tree of the utmost boundary. Nigh unto which is the Garden of Abode. When that which shroudeth did enshroud the lote-tree. The eye turned not aside nor yet was overbold. Verily he saw one of the Greater Signs revelations of his Lord" (LIII: 13–18, Pickthall edition).

The only other reference to the lote-tree is in the *sura *of the Event, namely the Day of Judgment, when those at Allah's right hand, that is, the faithful, will dwell "among thorn less lote-trees and clustered plantains, and spreading shade, and water gushing, and fruit is plenty" (Qur'an, LVI: 28–32, Pickthall edition).

Farooqi, in his book "*Plants of the Qur'an*" discusses at length the different names of the Qur'an's lotus tree: he suggests *Z. spina-christi *as an option, but on the other hand *Z. lotus *and *Z. spina-christi *are wild plants in Arabia. Another possibility he mentions is the Lebanon cedar (*Cedrus libani L*.), which is also called "*sidr *in Arabic. Farooqi concludes that the lotus tree of the Quran was indeed the Lebanon cedar, and the historical misunderstanding has perpetuated the mistaken name until the present day [8].

The continuing debate regarding the lotus tree in the Qur'an, not withstanding, the sacred tree found in the Middle East called *sidr*, the name given in the Qu'ran, is the Christ's Thorn Jujube (*Ziziphus spina-christi*).

#### Christ's Thorn Jujube in the medieval Levantine literature

Muslim as well as Christian pilgrims and travelers have described *Z. spina-christi *as a large tree that grew in the Land of Israel. The tree was usually recorded for its uses and as a symbol of holiness [13]. The pilgrims took branches of the tree back to their homeland as souvenirs in the belief that the Jesus's crown of thorns was made from such branches [20,21]. Estori ha-Parhi, for example, who visited the Land of Israel during the Mamluk period (13^th^–16^th ^centuries), wrote that the "*rimin*" is the "*nabaq*" in the language of Egypt and "*dum*" in the land of Canaan, and is also the tree named "*sidar*" [22].

In medieval medical literature the jujube appears frequently under various names, such as "*sidar*" or "*tsal*", while the fruit is called "nabaq" or *dum*" [14,23].

Clear-cut evidence of the medicinal uses and the economic value of the tree in the Land of Israel during the medieval period are found in Temple Mount documents: *sidar features *in a list of medicinal substances sold by the "*atarin*" (medicine vendors) in the markets of Jerusalem during the Mamluk period [24].

Al-Qazwini cites earlier authorities in stating that if the seeds are soaked in rose water and than planted, the fruits of the future tree will smell like roses. Similarly, if soaked in honey and milk, the future fruits will be sweeter and better [25].

In the past, various species of jujube grew in the Land of Israel that bore excellent fruit [26]. Al-Muqaddasi lists the tree among the widespread crops cultivated in the district of Falastin [27], and it was a popular food among the inhabitants of Tiberias [27]. Another source notes that the women of Egypt and the al-Sham region [Levant] used to comb their hair with the "*sidar*" [28].

#### Christ's Thorn Jujube as a useful plant in present day Levant

The fresh and the dried fruits of the plant are edible and highly valued locally by Arabs as well as Bedouins [17,29-32]. Bedouins collect and dry the fruits for future use in the winter, making a thick paste to be used as bread [17], a practice known already in ancient Egypt [2]. *Z. lotus L. *is similarly used in Cyprus [33] and in Arabia [34].

The wood is heavy and durable, and serves for artistic woodwork, while the branches and trunk are used as firewood and high quality charcoal [17,30-32].

### Ethnopharmacology of Christ's Thorn Jujube

The tree and its various parts have been an important source for pharmaceuticals since antiquity. Data on the medicinal uses of the plant are presented here in Table 1.

**Table 1:** Medicinal uses of Christ's Thorn Jujube

**Illness/uses**	**Parts and preparation**	**Region/ethnic group and reference**	**Historical references**	**World references**
Toothache, gum problems	Rubbing the teeth/gums with root powder (or bark)	Arabs, Bedouins [11].		Iraq [35]; Arabian peninsula [37].
Arthritis	Paste of crushed roots, leaves or branches; branches and leaves – inhale steam	Arabs, Bedouins [11, 36].		Arabian peninsula [37]. Dhofar [38].
General painkiller	Paste of crushed roots or branches and flour is applied	Arabs, Bedouins [11].		
Anodyne	Bark, leaves			Iraq [35].
Muscle pains	Branches and leaves – inhale steam	Sinai and Negev Bedouins [36, 39].		
Soothe pains	Leaves	Yemenite Jews [40].		
Bruises	Fruit, leaves, seeds			Arabian peninsula [37]. Dhofar [38].
Chest pains, asthma	Fruit, leaves, seeds		X Century [41].	Arabian peninsula [37].
Headache	Fruit, leaves, seeds			Arabian peninsula [37]. Dhofar [38].
Heart pains	Branches	Sinai and Negev Bedouins [36, 39].		
Eye inflammations	Powder of seeds, green leaves or roots as cataplasm.	Arabs, Bedouins [11]; Iraqi Jews [43].		Egypt (Bedouins) [42].
Stomach disorders: aches, constipation, heartburn.	Decoction of seeds leaves or fruit is drunk.	Arabs, Bedouins [11].	Ancient Egypt [2], (X Century [41, 45].	Iraq [35]; Morocco, [42]. Iberian Peninsula, 13^th ^century [46]. Dhofar [38].
Anthelmintic	Fruit, seed or leaf infusion	Arabs, Bedouins [11], Iraqi Jews [43].		Morocco, [42].
Hemorrhoids	Leaves	Yemenite Jews [40]; Iraqi Jews [43].	XIII Century [25].	
Diarrhea	Fruit or leaf infusion	Sinai and Negev Bedouins [36, 39], Yemenite Jews [40]; Iraqi Jews [43].	Ancient Egypt [2], X Century [41]; XIII [25].	Morocco, [42].
Increase milk production	Leaves boiled and liquid drunk	Sinai and Negev Bedouins [36, 39].		
Promoting pregnancy	Fruit – tea	Sinai and Negev Bedouins [36, 39].		
To ease prolonged labor	Leaves boiled and liquid drunk			Arabian peninsula [37]. Dhofar [38].
Wounds	Application of fruit juice	Iraqi Jews [43]; Arabs [11].	Ancient Egypt [2].	Dhofar [38].
Blisters	Fruit, leaves, seeds			Arabian peninsula [37].
Burns	Fruit – crushed and boiled	Iraqi Jews [43].		
Skin diseases and disorders	Boiled or crushed leaves, resin	Iraqi Jews [43].	X Century [41].	Arabian peninsula [37]. Dhofar [38].
Abscesses and furuncles	Cataplasm of boiled leaves			Morocco, [42].
Lung, chest and pectoral problems	Leaves or fruit	Iraqi Jews [43].		Iraq [35]; Arabian peninsula [37]; Iberian Peninsula, 13^th ^century [46].
Blood purifier and tonic	Leaves or fruit	Yemenite Jews [40].	Ancient Egypt [2, 44].	Iraq [35]; Arabian peninsula [37]; Dhofar [38];
High blood pressure	Leaves	Israel [47, 48].		Jordan [12].
Fractures	Cataplasm of boiled leaves			Arabian peninsula [37].
Emollient	Fruit or leaf infusion			Iraq [35]; Arabian peninsula [37]; Morocco, [42].
Depurative	Fruit			Iraq [35].
Cooling	Bark, leaves, fruit		Ancient Egypt [2, 44].	Iraq [35].
Tonic	Bark, leaves		Ancient Egypt [2].	Iraq [35].
Stomachic	Bark, leaves, fruit		Ancient Egypt [2, 44].	Iraq [35].
Measles	Fruit infusion			Morocco [42].
Febrifuge	Fruit infusion, resin		X Century [41].	Morocco [42].
Snake bites	Wood ash in vinegar		Leaves for bee or wasp stings, XIIIth century [49].	Morocco [42].
Astringent	Leaf infusion			Morocco [42]; Iraq [35].
Hair problems	Liquid from branches, fruit, leaves, seeds, resin.	Arabs [11]; Iraqi Jews [43].	X Century [41]; XIII Century [25].	Southwestern Saudi Arabia [50]; Arabian peninsula [37]. Dhofar [38].
Infant's powder	Powdered leaves			Yemen [37].
Colds	Fruit	Israel [47, 48].		Jordan [12].
Weight reduction	Fruit	Israel [47, 48].		Jordan [12].
Nervousness	Branches and leaves	Negev [36].		
Swollen organs	Fruit		Ancient Egypt [2].	
Diuretic	Wood		Ancient Egypt [2].	
Liver problems	Fruits		Ancient Egypt [2].	
Anus problems	Fruits		Coptic Egyptian Medicine [2].	

### Christ's Thorn Jujube as a sacred tree

An old Muslim legend tells about a Christ's Thorn Jujube that grows in Paradise and has leaves as many as there are human beings. Each leaf bears the names of a particular person and his or her parents. Every year, one day in the middle of the month of Ramadan, just after sunset, the tree is shaken. The names on the leaves that fall are of those who face death in the coming year. The process of the leaves' decay intimates the timing of their death; some leaves dry up and fall immediately while others wither slowly, signifying the time the person has left [51-53].

This legend reflects the respect in which Muslims hold all Christ's Thorn Jujube trees, wherever they are. No wonder that the tree has received so much attention in Arab folklore in the Land of Israel in the past, and continues to do so to the present day.

The Christ's Thorn Jujube is considered a sacred tree in Israel. When the tree reaches its 40 th year, the saints sit under it; therefore, the saints will destroy anyone who dares to cut down the tree or one of its branches. One story tells that "every Thursday evening the music of some instrument could be heard coming from some Christ's Thorn Jujube trees. Another story told and recorded in the Holy Land relates that lights were seen every Thursday night among the branches of few trees near "N'an'a" (Na'an) and 'Aqir" ('Aqron) [54,55].

The presence of saints under Christ's Thorn Jujube trees imparted their holiness to the tree, as happens with other species of trees. However, no other tree species are mentioned in the Holy Land as preferred by the saints.

Goldziher [56] sheds light on the importance and the great honor the Christ's Thorn Jujube has among the Arab population in the Holy Land. He cites the Abbé Barges [57] who described a large tree that grew in the garden of an Arab's house in Jaffa. This tree was treated with a special reverence by the local Muslims, who would hang colored cloths and lamps on it. The owner of the tree explained this kind of worship as the belief that the seeds from which the tree grew had fallen from the sky so the tree was sacred to the Prophet, who visited it at night. He added that all "good Muslims honored the tree". We assume that it was a Christ's Thorn Jujube since cloths and lamps were hung on its branches (*Z. lotus *is too low for this purpose), furthermore, Jaffa is far from the natural habitat of the lotus jujube.

We recorded a similar story in Kabul, a village in Western Galilee: "Lamps were lit every Thursday evening among the branches of the big Christ's Thorn Jujube tree which was in the village. Then the Sufi dervishes held their *zikr *ceremony (a special Sufic dance meeting) under this tree. They gathered there from various villages thereabouts, and the tree had its own sheikhs, who were not known in our village. The villagers were afraid to approach the tree, except for one old lady who would bring food and meat for the dervishes, as a gift from the local folk. The tree stood in the village until the 1950s (AĦmad Ţaha Yāsīn, Kabūl, 6 June 2004).

At times Christ's Thorn Jujube trees were used to mark the borders between estates of neighboring villages according to the common belief that the fence around Paradise was build of the wood of Christ's Thorn Jujube [55].

The special attitude to the Christ's Thorn Jujube in the Holy Land can be summarized and explained as the traditional belief that the tree should be esteemed and respected since it was probably the host of certain saints or other spirits [17]. In the words of Ţāhir 'Abu 'Antar (Ţamra, 14 June 2004), "The *sidr *tree is like a sheikh", and you have to pay it respect as you would elderly people.

In modern Islam, sitting under a Christ's Thorn Christ's thorn Jujube tree is considered lucky, since the Prophet saw such tree in Paradise [58]. This idea might underlie the traditional belief that a potion made of Christ's Thorn Jujube leafs is the best supernatural remedy to expel demons ('Ădil Abu Hamīd and Ibrāhīm QadaĦ, Kufr Manda, 3 June 2004; Abu Amīn Xāldi, Xawālid, 26 August 2004; Sāmya Hādi, Mazra'a, 24 August 2004, MaĦmūd Zir'ēni, 22 November 2004, Tur'ān).

In the village of Mġār several informants were recorded commenting on the fruit of two sacred Christ's Thorn Jujube trees, named after Sheikh Rabīs and Nabi Shu'eib: "You will never find worms [in the fruit of these two trees], as you do in the fruit of other similar trees" (i.e., of this species). This was due to the sacredness of these two trees, which were blessed (Muhra Bahajāt, Qāsim Fādhil, Şalah Fādhil, Mġār, 19 May 2001). According to another informant (Ġāsam MuĦammad 'uqabi, Ţūba-Zanġariyya, 16 June 2004), "The fruit of the Christ's Thorn Jujube was eaten by Muslim fighters of the early Islamic period; therefore the tree is honored and may not be uprooted".

It is common, then, to find Christ's Thorn Jujube trees serving as sacred trees in many villages and at sheikhs' graves all over the Holy Land, but mainly in Upper Galilee (Sheikh Rabīs and Nabi Shu'eib, Mġār, Rabbi Avdimi grave in Haifa (cut at 2003), Sakhnīn, Sheikh Radwān near Nahariya, Sajarāt al-'Arūsa near Kābri).

In the Negev, barren women had to make a pilgrimage to a sacred Christ's Thorn Jujube [36].

### Christ's Thorn Jujube proverbs

Demons (genies) avoid the Christ's Thorn Jujube tree because of its sanctity, and this precisely is why it is "good" to sleep under it (MuSţafa Kamirat, Ibtin, 13 January 2003; Ibrāhīm QadaĦ, Kufr Manda, and 3 June 2004; 'Ali Sulaymān Xuţba, 'Arrābe, 6. June 2004; Yusuf Nimmr Masar, Sakhnīn 1 January 2005). The following Arab proverb signifies that Christ's Thorn is blessed while the Carob tree (*Ceratonia siliqua *L.) is considered cursed: "innōm biĦlu taĦt iddōm, innōma taĦt ilxarrūbe mush marghūbe", which means "the sleep under Christ's Thorn Jujube tree is sweet and the sleep under Carob tree is not desirable" [59] and thirteen informants in our survey. The Carob tree is associated with a bad luck, and sitting under it is considered dangerous, especially at night since the tree is a dwelling place for bad spirits. The red color of the leaf petioles, which resembles blood, is a sign of bad luck as well [17].

One explanation for why the Carob is cursed is this: "There is always a chance of finding a snake in the trunk and therefore you may not sleep under it; bees dwell in its trunk and branches as well" ('Ali Khalil Musa Kna'ane, 'Arrābe, 6 June 2004; 'Ali Sulaymān Xuţba, 'Arrābe, 6 June 2004: Yusuf Nimmr Miser, Sakhnīn 1 January 2005). Another informant, Ibrāhīm QadaĦ (Kufr Manda, 3 June 2004), added more information regarding the dangers sleeping under the Carob tree: "The demons gather under Carob trees and every once in a while God punishes them by striking them with lightning. These trees are big, and attract lightning anyway". Other informants reported that snakes and demons like the Carob tree, and therefore it is cursed; people who slept under this tree went mad (Atef Mansur, Kaukab Abu-el-Heija, 13 May 2003; Hassan Jadir, Bir El Maksur, 30 December 2003; Nassr Khalil, Sakhnīn, 1 January 2005). One informant recounted "The Carob is a bad tree, the snakes that dwell in its branches hurt people. However, snakes that dwell in the Christ's Thorn Jujube will never harm a human being" (Sāmya Hadi (Mazra'a, 24 August 2004). Because of their black color, the Fig tree and the Carob tree are both considered cursed and the cause of misfortune [54]. The following proverb expresses this feeling: " Xarrūbe wa ttīn – maskanat iššayaţīn", means "Carob and Fig tree – (are) Satan's residency" (Abu-Amin Xāldi, Xawālid, 26 August 2004; 'Ali Khalil Musa Kna'ane, 'Arrābe 6 June 2004). Canaan likewise explains that both trees (Carob and Fig) bear black fruits, and they are the preferred dwelling places of demons. The genies gather under them for their meetings and nightly parades [59].

Additional explanations of why it is "good" under the Christ's Thorn Jujube are these: "The Christ's Thorn Jujube is a tree from heaven and therefore it is good to sleep under it" (MuHammad Ţāher, Rummāna, 22 November 2004; Abu-Razz, Bu'eina-Nujeidāt, 16 August 2004; Yusuf Nimr Masar, Sakhnīn 1 January 2005); "In arid zones the Christ's Thorn Jujube is the only shady tree and therefore it deserves a special attitude" (Raja Xaţīb, Deir Ħanna, 2 August 2004).

### Christ's Thorn Jujube and animism

Special honor is given to the Christ's Thorn Jujube in Iraq, even more than to the Palm. Uprooting of a tree that has already fallen is considered a sign of impending disaster; should a man cut down a Christ's Thorn Jujube tree, he will soon fall ill and die. The tree is thought to groan when it cut and its sap, red as blood, gushes out of the slashed trunk, justifying the idea that the tree has a life similar to a human's [60].

The belief that blood flows from trees organs has ancient roots. Ovid [61] tells of Erysichthon, king of Thrace, who commanded that a sacred oak tree dedicated to Demeter be cut down. The appeal of the Dryad that lived in the tree was in vain. The tree was chopped down, and she was doomed to die with the demise of her abode; Demeter's revenge was immediate and singularly cruel. The king was condemned to an eternal unsatisfied hunger.

The tradition of regarding the punishment of whoever touches a sacred tree universal, and it has been one of the main characteristics of tree worship throughout history. Stories about groaning or bleeding trees are common [62-64].

Among the Bedouins of the Negev (southern Israel), a similar tradition is known: the red sap drops that flow out of Christ's Thorn Jujube trees gives them a human essence because of its resemblance to blood. It is a short step from these phenomena to tree worship, animism and the worship of the saint's spirit that dwells in the tree [65].

The old people of Mġār used to tell about the Christ's Thorn Jujube tree of Nabi Shu'eib (see above) that bled when it was cut; the children of the village, being skeptical, would make small cuts in the tree to see if it really would bleed ('Issa Sakrān, Mġār, 16 June 2004).

A similar story explaining the sacredness of Christ's Thorn Jujube tree was told in the Negev in 1977:

"In a place where we used to live a long time ago, in the southern part of Israel, there was one Christ's Thorn Jujube tree (*sidr*) under which stains of red sap, similar to blood, were found every morning... The women would hang white pieces of cloths on [the tree], the men did not cut it, and the tree grew to be a very big, even bigger than the tent we sit in". The sacredness of the tree gushes from the presence of the spirits of the dead people that dwell there. The tradition is that Christ's Thorn Jujube trees are sacred wherever they are... The Bedouins' explanation for this phenomenon usually concerns the holy man or holy people that dwell in or under the tree [65].

In Morocco a story was recorded about a tree named after Sides Bumhadi. A man climbed up it to cut some branches, and a copious flow of blood, as if fifty bulls had been slaughtered, came out of the tree. The terrified man jumped down and stayed there stunned [66].

In Iraq women occasionally visited Christ's Thorn Jujube trees. They lit straw torches under the tree and put incense on charcoal. Before departing they would leave four lighted candles on the ashes. This ceremony was to heal sick family members [60].

Lighting candles and other ceremonies for healing people are typical to tree worship in the Muslim and the Christian world alike, among the different ethnic groups in Israel (our observations), and all over the world [67,68].

As part of the ceremonies in Iraq, green clothing were hung on the branches of the Christ's Thorn Jujube tree, and sometimes even offerings of food were left under it [60].

The custom of hanging clothes on trees in general and sacred trees in particular is world-wide. The idea is that the disease is transferred from the sick person's clothes to the tree [69]. Green clothes are used especially in the Muslim world, because the color green is sacred in Islam [70].

Other evidence of respect for Christ's Thorn Jujube trees in Islam is the following custom. In Iran [52], Iraq [60], India [71], and southwestern Saudi Arabia [50] the bodies of dead Muslims were washed with water in which Christ's Thorn Jujube leaves had been soaked. The purpose was to preserve the body and satisfy the angels [52]. A similar usage was recorded in Israel by one informant [11], and others described this as custom that was practiced in Israel in the past, and although rarely, even today ('Ădil Abu Ħamīd and Ibrāhīm QadaĦ, Kufr Manda, 3 June 2004; Abu Rāzi, Bu'eina-Nujeidāt, 16 August 2004; Sheikh AĦmad Abu 'Umar, Mjd ilKrūm, 28 June 2004).

### Customs related to the tree

The grave of Sheikh ŞalāĦ, which is in the old Muslim cemetery 400 meters east of the al-Jazzār Mosque in Acre (Jehoshafat Street), is prominent mainly because of its green domed roof. Ten meters south of the building a huge Christ's Thorn Jujube tree once grew. Its trunk was a meter in diameter and the tree shaded many graves. But its roots caused damage to a few graves so it was cut down at April 2000. Many iron nails were found on the cut trunk, arranged in groups of three. Māhir Zahra (Acre, 12 April 2002), a researcher on the history of Acre, described and explained this phenomena:

"Women who felt the need to break an evil eye spell would conduct the following ceremony. The woman would come purified, a few days after her period, on Friday early morning before the call of the muezzin, and approach the tree. She was not allowed to talk to anybody from the time she had awoken that day. She had to wait near the Christ's Thorn Jujube tree with an hammer and nails; after each call of 'Allahu 'akbar, she had to drive a nail into the tree – three times in all".

In this cemetery the sounds from the Mosque were heard very well. The nails were hammered into exorcise the woman or one of her family members of the evil eye. She had to remain quiet and was not allowed to talk all the way back home. Then she had to wash again and purify herself and go to sleep.

This tradition has to do with the Suffis who arrived in Acre at the beginning of the 18^th ^century from North Africa. The citizens of Acre have known of the tree and the nails tradition since the middle of the 18^th ^century. According to 'Araf [72], "Such nails can be seen in many other old trees found near saints' graves, or sacred trees, such as the trunk of the tree in the Druze village Jat in the upper Galilee. This tree is named Sajarat Abu 'Arus. Yet when we visited this specific tree (April 2003) we found no nails in it at all! Occasionally nails were seen in the trunks of sacred trees, but these were usually species of oak and the nails did not appear in triplets.

Similar customs have been recorded in other countries in the Middle East, India, and Europe [62,64,73-75].

Old women in the village of Ţamra, Western Galilee, used to tell of a custom of hammering nails into big old olive trees to protect against the evil eye. They called them "nails in the eye of the devil" (AĦmad Ţaha Yāsīn, Kabūl, 14 June 2004).

The purpose of this custom was twofold: to "stick" someone to the sacredness by attaching him or her to the powers of the tree and to expel demons with the tree's help ('Ădil Abu Ħamīd. Kufr Manda, 3 June 2004).

An interesting piece of medieval evidence of a similar custom appears in the Cairo Genizah (14^th ^century): "Beautiful trees around the grave, they smell good and nails are stuck in them" [76].

Hammering nails and hanging cloths are "tying" rituals, whereby the person seeks healing or a solution to problems by transferring his or her illness or problems to the tree, or to whatever object the cloths are hung on or nails hammered into. Such "tying" is one of the best known and commonest belief practices all over the world among Christians, as well as among Muslims and their predecessors in the Middle East [53,64,70,77]. This tradition still exists today in Europe (Belgium) [78]. In Egypt, nails driven into tree trunks signify the prayers of the believers. People come to sheikhs' trees to be cured of headache or other ailments. In asking the sheikh for help they hammer nails into the trunk and wind some of their hair around the nails [79]. A ceremony of this kind was recorded at sacred graves in Turkey [64].

In England (Cornwall) and Germany (Oldenburg), believers used to hammer nails into tree trunks "where the sun cannot reach" to heal toothache [80]. Variations of this were seen in Nepal and India (Samir Ţafiš, Beit Jan, 18 March 2002) and in Turkey [64,81].

In India the Emetic nut tree (*Strychnos nux-vomica *L.) is considered the prison of all demons. Occasionally such trees can be seen with trunks full of nails as a precaution against demons. If a demon or bad spirit dares to attack a human, the exorcist forces it back into the tree with a nail. With each driven nail the demon declares that it will not attack again. Nailing the demon into the tree trunk is the best way to give it a life sentence [82,83].

Sacred trees in the West Himalayan region are the object of a similar custom: travelers hammer nails into the trunk when passing by as a protective step against diseases, death, and any damage to their sheep, cattle, or crops. The explanation for this act, according to traditional beliefs, is that it dispels evil powers [84].

A square in central Vienna is named "Stock am Eisen", which means literally "iron on the stick". A glass case stands on one of the corners of the square containing a replica of a piece of wood in which some nails are stuck. A known tradition from the 16^th ^century tells that any apprentice who completed his duties in the town would hammer a nail into a tree that grew in the square for good luck [73,85]. Some are of the opinion that this was a gypsy tradition introduced from India [74].

Returning to Acre, cutting down the "nails tree" in the city apparently entailed the sad result of the extinguishing a unique and rare custom in Israel. This ritual in Israel and many others concern sacred trees, were evidently a link in the cultural chain between India and Europe.

### "Holy" vs. "Sacred" trees

Simmons [86] distinguishes tree rituals wherein a certain species of tree is considered holy, as in the case of the sacred fig [Bo tree] (*Ficus religiosa *L.), from ritual in which individual trees are sacred because of special characteristics or have won respect through their location in a holy place or their association with a holy person [86].

The veneration of trees in Israel mainly stems from events with saints that occurred near them, so different tree species, were sanctified. In Iraq, by contrast, the Christ's Thorn Jujube tree is worshiped because this tree is mentioned in the Qur'an.

Most trees belong to the first group above, so their botanical species is not relevant. We apply the adjective "sacred" to these trees; we use the epithet "holy" for trees that have gained special respect because their botanical species is part of a religious ritual.

As far as we know, in the Land of Israel, all known admired trees are "sacred", having won honor because of their physical location near saint's graves or their connection to the deeds of religious, military, or other admired figures [64,79,87]. We are not aware of any "holy" trees.

One citation we recorded from an informant sums up the matter: "The sacred trees are the tombstones – the memento of a saint or holy man, so their importance is relative to the man holiness. The holier the saint, the more sacred the tree" (Sa'īd MaĦamūd, YānuĦ, 25 May 2003).

Another explanation we recorded has this variation: "Blessed trees are memorials to unique figures in the Druze history and religion; because the Druze tradition forbids any tomb sign or offerings, special people are remembered by big sacred trees" (Sheikh Šāhīn Ħusayn, Beit Jan, 12 September 03).

### Christ's Thorn Jujube tree in the Druze tradition

Among the Druze, "sacred" trees are called "blessed" because according to their tradition only humans can be sacred. However, sacred figures can transfer some of their special powers to a tree. God's blessing is thought pass to the holy man and is then transferred to the blessed tree [70].

The holiness of the Christ's Thorn Jujube tree among the Druze is a tradition beginning in Islam (it is the tree of the seventh heaven). When the prophet ascended to Paradise he reached the seventh heaven and the last tree, named "*Sidrat al-Muntaha*" (the *sidr *tree of the last frontier).

The Druze argues that the last Christ's Thorn Jujube tree symbolizes a certain figure and the story is about an imaginary spiritual trip in which the "*sidr *signifies an important and admired figure (ŞalāĦ Xaţīb, Mġār, 4 October 2003).

On the main road to Nabi Shu'eib, one of the holiest places for Druze in Israel (believed to be the grave of the prophet Jethro); there is a huge Christ's Thorn Jujube tree. In the past this important tree served as a meeting point for pilgrims before approaching the holy place. Whoever arrived first waited for the others under that tree. Over the years the tradition of the first meeting point took root, and the tree became a station for praying as well. When the pilgrims reached the tree they became very excited, and this is how the tree was named "Sidrat Nebi Shu'eib" (Abu Jalāl Qizl, ŞalāĦ Xatib and 'Ali 'Araydi, Mġār, 19 May 2001). The tale says that this tree grew out of a runner of a Christ's Thorn Jujube tree named "Sheikh Rabis (a huge "blessed" Christ's Thorn Jujube tree that grows in the middle of Mġār, two kilometers away from this tree). The person Sheikh Rabis (to whom the tree is dedicated) was a Muslim, but Druze's and Christians admire him as well. One informant explained: "As the Druzes came out of Islam, the Druzes' blessed tree came out of the Muslim one" (ŞalāĦ Xaţīb, Mġār, 4 October 2003).

## Conclusion

The Christ's Thorn Jujube tree is widespread in various parts of the Land of Israel. In fact, its distribution is very broad and goes beyond desert areas. It also grows along the coastal plain and around Jerusalem. Throughout history, but mainly in the Middle Ages, the tree was seen as sacred [74] and was used for food [27]. Evidence of medicinal use of the Christ's Thorn Jujube in our region is very rare; we were able to trace one source, a list of substances sold in Jerusalem by medicine vendors in the Mamluk period (14^th^–15^th ^century) [24]. The current medicinal uses in Israel are the same as in other parts of the Middle East (Table 1). We are unable to show any indication that some uses are endemic or more prevalent in one or another ethnic group.

Tree worship is deeply rooted in human culture and attitudes to the Christ's Thorn Jujube are a good example of these traditions. Eliade has taught us about the important religious role these trees play in different cultures. In almost every culture trees are presented as symbols of life, ongoing fertility and eternity, as well as resurrection. Trees are not admired for themselves, but because of what was discovered through them, what they symbolized and expressed [89]. Canaan puts a similar argument: "The villagers (of the Holy Land) do not admire the trees, but the divine powers acting through them and derived from the imaginary figure of God, which his soul is probably still in a temple, cave or spring to which the trees are connected. Occasionally the holy man is revealed in a tree or around it" [59].

Frese and Gray [90] emphasize that trees are an embodiment of nature, which represents the holy continuity of spiritual worlds, cosmic as well as physical. The tree sometimes symbolizes divinity or another holy entity or even a holy object.

Tree worship was prevalent in pre-Islamic pagan Arabia. Trees were frequently considered the abode of genies. The pagan Arabs associated divine characteristics with certain trees and worshiped them through special rituals, which included the hanging of colored cloths, ornaments, and weapons on their branches [58,91,92].

When the new religion of Islam evolved a war of annihilation was waged between the new Muslims and the pagans. This war involved the cutting down of sacred trees by the Muslims and strict prohibition against any form of tree worship [52,56].

This short review shows that tree worship prevailed in Arabia in the pre-Islamic period. Despite the battle against the pagan practice, some of the ancient rituals survived and still prevail in the Middle East [70].

The Christ's Thorn Jujube tree won unique honor because of a mention in the Qur'an; however, this species enjoys no special significance, botanical uniqueness, or exceptional size (compared with the great oak trees in Europe) which form the basis for tree worship in pagan Europe, including the Greek and Roman cultures) [93,94].

Most of the known traditions and rituals that were recorded in relation to the Christ's Thorn Jujube in the Middle East, such as candle lighting, incense burning, cloth hanging, and nail hammering, are known to be part of worshiping sacred trees in Israel and around the world. A similar case is the supernatural powers of the sacred tree and the fear of punishment as a result of cutting down the tree or dishonoring it. These customs do not relate to a particular tree species and are not found in Islamic lands alone.

The supernatural qualities attributed to the Christ's Thorn Jujube, such as blood flowing in its veins, sounds it makes when it is cut, and its being the abode of a saint's spirit or ancestors, are well documented in the literature of sacred trees.

The custom of washing the dead with leaves of the Christ's Thorn Jujube seems unique to this species and to Islam; we could not trace any similar custom elsewhere. We would like to suggest that this might be evidence of the uniqueness of the Christ's Thorn Jujube tree.

In light of the distinction between "holy" and "sacred" trees, we maintain that the Christ's Thorn Jujube is the closest species to a holy tree in Israel and the Middle East, as well as being a sacred tree. The holiness originated with the citation in the Qur'an, and the sacredness arose from events in the lives of saints, heroes, and holy men that happened near the trees.
